# Doxorubicin-Induced TrkAIII Activation: A Selection Mechanism for Resistant Dormant Neuroblastoma Cells

**DOI:** 10.3390/ijms231810895

**Published:** 2022-09-17

**Authors:** Lucia Cappabianca, Michela Sebastiano, Marianna Ruggieri, Maddalena Sbaffone, Veronica Zelli, Antonietta Rosella Farina, Andrew Reay Mackay

**Affiliations:** Department of Biotechnological and Applied Clinical Sciences, University of L’Aquila, 67100 L’Aquila, Italy

**Keywords:** neuroblastoma, doxorubicin, doxorubicin-resistance, TrkA, TrkAIII, tyrosine kinase inhibitors, ryanodine receptors, Ca^2+^ uniporter, calmodulin, Hsp90, cyclic hypoxia

## Abstract

Patients with advanced neuroblastoma (NB) receive multimodal clinical therapy, including the potent anthracycline chemotherapy drug doxorubicin (Dox). The acquisition of Dox resistance, however, is a major barrier to a sustained response and leads to a poor prognosis in advanced disease states, reinforcing the need to identify and inhibit Dox resistance mechanisms. In this context, we report on the identification and inhibition of a novel Dox resistance mechanism. This mechanism is characterized by the Dox-induced activation of the oncogenic TrkAIII alternative splice variant, resulting in increased Dox resistance, and is blocked by lestaurtinib, entrectinib, and crizotinib tyrosine kinase and LY294002 IP3-K inhibitors. Using time lapse live cell imaging, conventional and co-immunoprecipitation Western blots, RT-PCR, and inhibitor studies, we report that the Dox-induced TrkAIII activation correlates with proliferation inhibition and is CDK1- and Ca^2+^-uniporter-independent. It is mediated by ryanodine receptors; involves Ca^2+^-dependent interactions between TrkAIII, calmodulin and Hsp90; requires oxygen and oxidation; occurs within assembled ERGICs; and does not occur with fully spliced TrkA. The inhibitory effects of lestaurtinib, entrectinib, crizotinib, and LY294002 on the Dox-induced TrkAIII and Akt phosphorylation and resistance confirm roles for TrkAIII and IP3-K consistent with Dox-induced, TrkAIII-mediated pro-survival IP3K/Akt signaling. This mechanism has the potential to select resistant dormant TrkAIII-expressing NB cells, supporting the use of Trk inhibitors during Dox therapy in TrkAIII-expressing NBs.

## 1. Introduction

Neuroblastomas (NBs) are highly heterogeneous, aggressive pediatric tumors that arise from embryonic sympathoadrenal neural crest cells and are usually diagnosed at a late stage. Despite the use of intensive multimodal clinical treatment strategies, the prognosis of advanced NB remains poor, and the acquisition of treatment resistance leads to a high frequency of post-treatment relapses. In fact, the a 50% recurrence rate significantly reduces the survival time, making the effective cure of advanced NB a major challenge [1,2,3].

Intensive multimodal clinical treatment strategies for unfavorable, high-risk, advanced NB include an induction phase with alternating high-dose chemotherapeutic agents, including the potent quinone–anthracycline antibiotic doxorubicin (Dox) [4,5]. However, the clinical response to Dox is limited by drug resistance and cardiotoxicity, which underlie frequent treatment failure, post-treatment relapse, and disease progression [4,5,6,7]. Therefore, identifying and inhibiting the mechanisms responsible for Dox resistance and cardiotoxicity is critical if improved efficacy is to be achieved.

The occurrence of Dox cytotoxicity depends upon multiple mechanisms, including DNA and RNA intercalation; the inhibition of topoisomerase II leading to DNA double-strand breaks; and ROS formation leading to cytotoxic damage to lipids, proteins, and DNA [8,9]. In addition, Dox inhibits cancer cell proliferation by activating the DNA damage response components ATR, p53, Chk1, and Chk2, resulting in p53- and CREB3L1-dependent p21/WAF1 expression and inhibitory Src phosphorylation, which promote G1/S arrest and senescence [10,11,12].


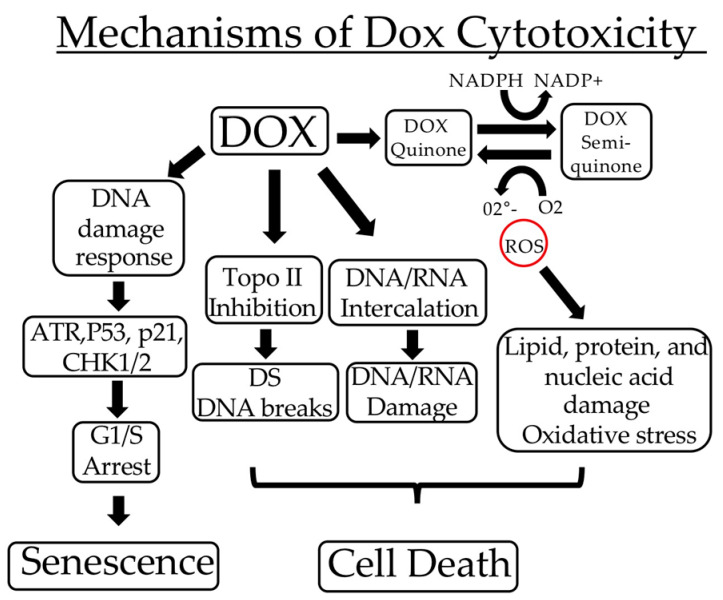


Dox resistance has also been attributed to multiple mechanisms [5,13,14,15,16]. In NB cells, Dox resistance is associated with a cancer stem cell (CSC)-like phenotype reflecting increased expression of ABCG2 and ABCG3 drug efflux pumps and survival-promoting Akt and JARID1B histone lysine demethylase signaling. This implies that conditions and signals that promote NB CSC formation also promote Dox resistance [17]. Dox-induced Akt activation is a major component of NB cell survival signaling, and Akt inhibitors enhance NB cell responses to Dox [18,19,20,21,22]. Akt exerts its pro-survival effects through multiple mechanisms, including the inhibition of Fas ligand expression, the induction of BAD phosphorylation leading to Bcl-xL release, the inhibition of pro-apoptotic caspase-9, glycogen synthase kinase 3-β [18], and the ZAK/MKKK4/MKKK7/JNK module [20,22]. In addition, Dox-resistant NB cells secrete factors that activate pro-survival STAT3 and Akt signaling in neighboring Dox-sensitive cells [18,22,23]. NB cells also exhibit dox-induced pro-survival Src signaling in association with an impaired p53 function [24]. Dox resistance in NB cells is also reduced by histone deacetylase and JAK2 inhibitors [25,26]. The combination of Dox resistance and Dox-induced senescence promotes tumor cell dormancy and stemness, increasing the potential aggressive post-therapeutic relapse [12].


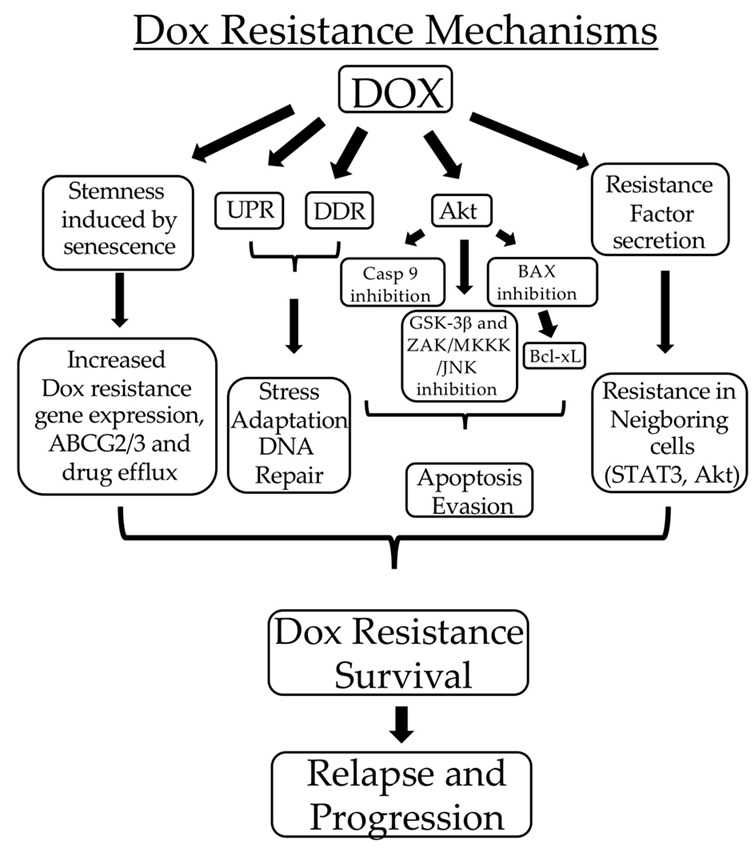


We previously reported that recurrent and metastatic advanced-stage NBs show enhanced expression of the oncogenic alternative TrkAIII splice variant of tropomyosin-related kinase A (TrkA). This identifies alternative splicing as a mechanism by which TrkA NB-suppressing signals can be converted to oncogenic signals [27,28]. TrkAIII mRNA is characterized by in-frame skipping of exons 6, 7, and 9, and TrkAIII is expressed as a variant protein that does not contain the extracellular D4-IG-like TrkA domain, encoded by exons 6 and 7. In fully spliced TrkA receptors, this domain is required for cell surface receptor localization, the prevention of ligand-independent activation, and optimized ligand binding [27,28,29,30]. In contrast to fully spliced TrkA, TrkAIII is not expressed at the cell surface, but accumulates in intracellular pre-Golgi membranes. In this intracellular context, TrkAIII exhibits spontaneous, cell-cycle-regulated, ligand-independent activation, resulting in survival (IP3-K/Akt, Bcl-xL, and Mcl-1), proangiogenic (MMP-9 and VEGF) and antioxidant (SOD2) gene expression, and signaling associated with a CSC-like phenotype [27,28]. Alternative TrkAIII mRNA splicing in NB cells is enhanced by agents that promote severe ER stress, the SV40 polyoma virus large T antigen, and the hypoxia mimic CoCl_2_ [27,28]. TrkAIII transforms NIH3T3 cells and promotes the primary tumorigenic and metastatic activity of NB models, confirming an oncogenic function [27,28].

The therapeutic potential of targeting TrkAIII in NB has recently been demonstrated in a report of the durable and persistent (>5 years) response to compassionate entrectinib Alk/Trk inhibitor therapy in an infant with refractory, relapsed, metastatic, TrkAIII-expressing NB, in the context of exhausted treatment options [31]. This has improved the future prospects for expanding the Trk inhibitor therapy—approved for Trk fusion oncogene-driven cancers—to refractory, advanced, metastatic NBs expressing TrkAIII.

In the present study, we identify a novel function for TrkAIII in Dox resistance, characterized by Dox-induced TrkAIII activation in NB cells rendered senescent by Dox-induced cell cycle arrest. This novel mechanism has the potential to select resistant, dormant, TrkAIII-expressing NB cells that could re-enter the cell cycle with a more metastatic phenotype, supporting the use of clinically approved Trk inhibitors.

## 2. Results

### 2.1. Dox Induces TrkAIII Activation in Non-Proliferating TrkAIII SH-SY5Y NB Cells

**Figure 1:**
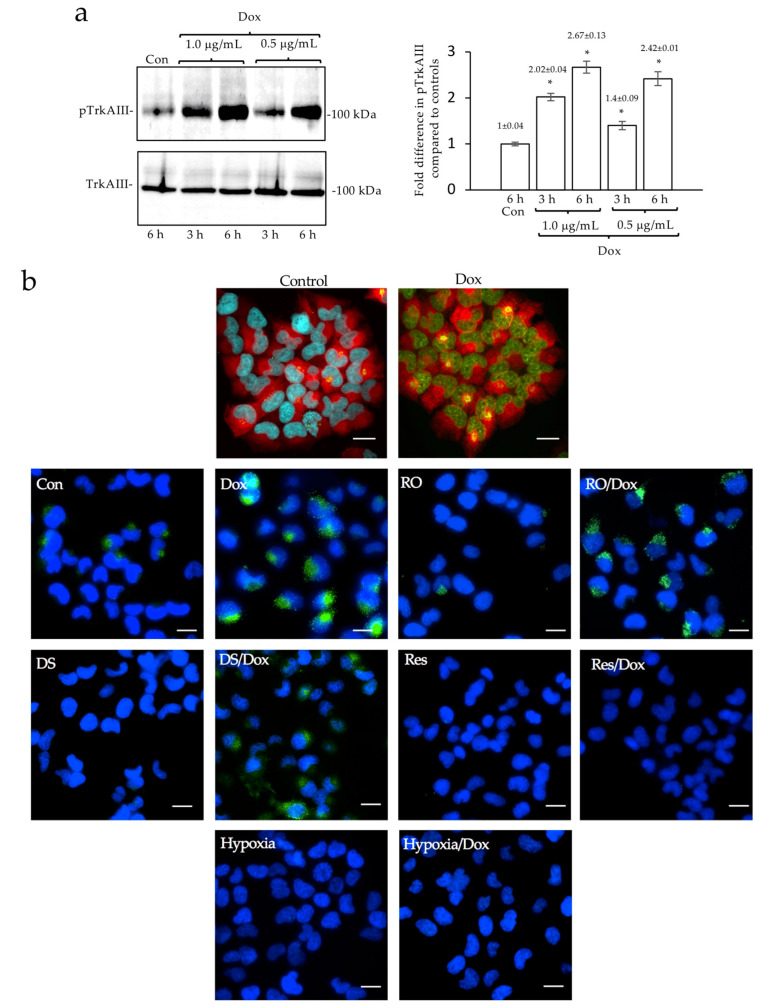
*Dox induces TrkAIII activation.* (**a**) Representative Western blots and accompanying histograms, demonstrating an increase in constitutive TrkAIII phosphorylation levels in TrkAIII SH-SY5Y cells (Con), following treatment with Dox, at the concentrations and times indicated (* = statistical significance). (**b**) Indirect IF micrographs, demonstrating increased TrkAIII phosphorylation (yellow) overlapping TrkAIII (red) in Dox-treated (1 μg/mL for 6 h) (right) compared to untreated (left) TrkAIII SH-SY5Y cells, plus indirect IF comparisons in TrkAIII phosphorylation (green) in TrkAIII SH-SY5Y cells either untreated (Con) or treated for 6 h with Dox (1 μg/mL) (Dox); RO-3306 (10 μM) (RO); RO-3306 (10 μM) plus Dox (1 μg/mL) (RO/Dox); DS16570511 (40 μM) (DS); DS16570511 (40 μM) plus Dox (1 μg/mL) (DS/Dox); resveratrol (100 μM) (Res); resveratrol (100 μM) plus Dox (1 μg/mL) (Res/Dox); hypoxia (<0.1% O_2_); hypoxia (<0.1% O_2_) plus Dox (1 μg/mL) (Hypoxia/Dox) (Bar = 10 μm).

In the time course, dose–response experiments, the Western blots detected a significant increase in phosphorylated compared to total TrkAIII levels in TrkAIII SH-SY5Y cells treated with Dox for 3 and 6 h (*p* = 0.039 and 0.0105 for 0.5 μg/mL Dox, and *p* = 0.0089 and 0.0139 for 1 μg/mL Dox, respectively, df = 2) (Figure 1a). Enhanced intracellular TrkAIII phosphorylation overlapping with the TrkAIII expression was also detected by indirect IF following Dox treatment for the time and concentration indicated in the figure legend (Figure 1b).

**Figure 2:**
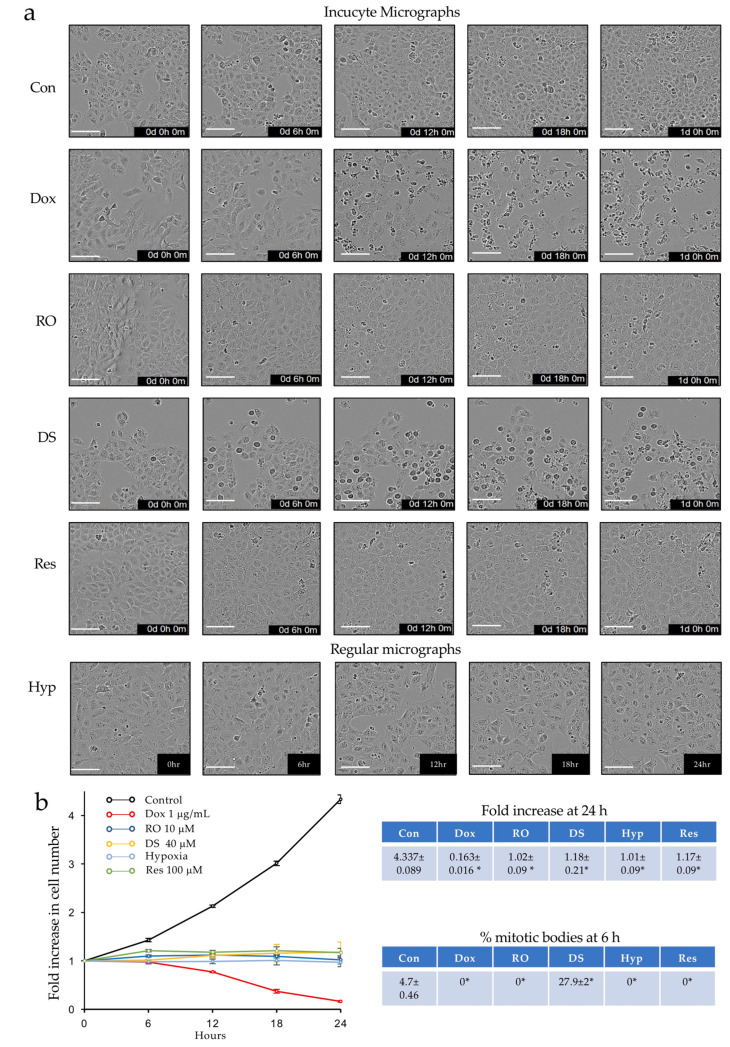
*Effects of Dox, RO-3306, DS16570511, resveratrol, and hypoxia on TrkAIII SH-SY5Y proliferation.* (**a**) Representative IncuCyte and regular micrographs of TrkAIII SH-SY5Y cells, untreated (Con) or treated with Dox (1 μg/mL) (Dox), RO-3306 (10 μM) (RO), DS16570511 (40 μM) (DS), resveratrol (100 μM) (Res), and hypoxia (<0.1% O_2_) (Hyp) for the times indicated. (**b**) Line graph demonstrating differences in fold increases in live cell numbers, TrkAIII SH-SY5Y cells, untreated (Con) and treated with Dox (Dox), RO-3306 (RO), DS16570511 (DS), hypoxia (Hyp), and resveratrol (Res), at the concentrations and times indicated, plus tables reporting the mean (±se) fold increase in live cell numbers at 24 h and the percentage of mitotic bodies at 6 h in untreated TrkAIII SH-SY5Y cell cultures (Con) and following treatments with Dox, RO, DS, Hyp, and Res at the above concentrations (* = significantly different to controls) (bar = 100 μm).

In the proliferation assays, the untreated TrkAIII SH-SY5Y cells exhibited a doubling time of ≈12 h, which translated into a ≈4-fold increase in cell numbers at 24 h (Figure 2a,b). The Dox completely inhibited the TrkAIII SH-SY5Y proliferation and reduced the live cell numbers by ≈84% at 24 h, with the cell numbers failing to increase throughout the 24 h time course (Figure 2b). The TrkAIII SH-SY5Y proliferation was also completely blocked by the CDK1 inhibitor RO-3306 [32], the Ca^2+^ uniporter inhibitor DS16570511 [33], the antioxidant resveratrol [34], and hypoxia, all of which prevented a significant increase in cell numbers throughout the 24 h time course (Figure 2a,b).

The evaluation of the number of cells in overt mitosis at 6 h revealed that compared to untreated TrkAIII SH-SY5Y controls, the TrkAIII SH-SY5Y cells treated with Dox, RO-3304, resveratrol, and hypoxia exhibited a complete absence of mitotic bodies. In contrast, cells treated with DS16570511 exhibited a ≈7-fold increase in mitotic bodies, consistent with DS16570511-induced M-phase arrest [35] (Figure 2b).

**Figure 3:**
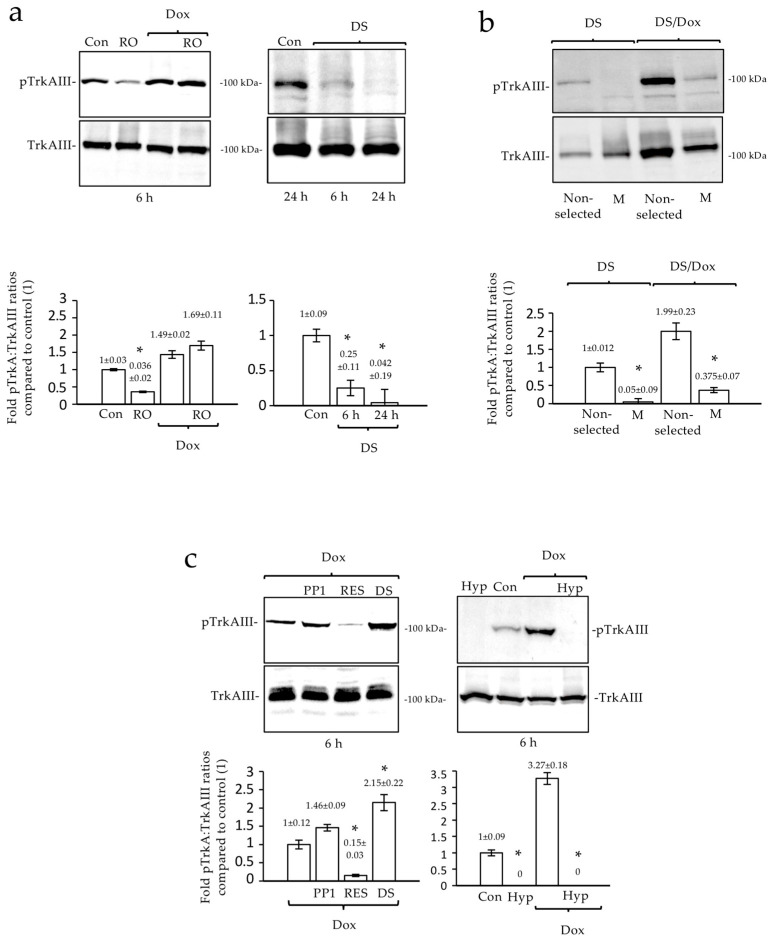
*Dox-induced TrkAIII activation occurs in cells arrested in proliferation but not the M phase and is oxygen- and oxidation-dependent*. Western blots and histograms demonstrating the following: (**a**) a reduction in TrkA phosphorylation relative to total TrkAIII in TrkAIII SH-SY5Y cells treated with RO-3306 (10 μM) (RO) alone but not RO-3306 (10 μM) plus Dox (1 μg/mL) (RO/Dox) and following treatment with DS16570511 (40 μM) for the times indicated; (**b**) differences in TrkAIII phosphorylation in detachment-enriched M-phase TrkAIII SH-SY5Y cells induced by DS16570511 (40 μM for 24 h) compared to non-selected DS16570511-treated counterparts, and in Dox (1 μg/mL, 6 h)-treated enriched M-phase TrkAIII SH-SY5Y cells compared to the Dox-treated, non-selected counterparts; (**c**) reduction in Dox (1 μg/mL)-induced TrkAIII phosphorylation by resveratrol (100 μM) but not by PP1 (1 μM) or DS16570511 (40 μM) and inhibition of constitutive (Con) and Dox (1 μg/mL)-induced TrkAIII phosphorylation by hypoxia (<0.1% O_2_) (Hyp) at the times indicated in TrkAIII SH-SY5Y cells (* = statistical significance).

In experiments to further interrogate Dox-induced TrkAIII activation in non-proliferating TrkAIII SH-SY5Y cells arrested by the CDK1 inhibitor RO-3306 [36], the Western blots detected a significant reduction in phosphorylated TrkAIII levels (*p* = 0.0032, df = 2) (Figure 3a). In contrast, RO-3306 did not inhibit but rather increased the TrkAIII phosphorylation in Dox-treated TrkAIII SH-SY5Y cells, which were already significantly enhanced by the Dox treatment alone (*p* = 0.006, df = 2) (Figure 3a).

In the TrkAIII SH-SY5Y cells treated with DS16570511, the Western blots also detected a significant reduction in phosphorylated TrkAIII levels at 6 h (*p* = 0.0341, df = 2) and 24 h (*p* = 0.045, df = 2) (Figure 3a). In contrast, the phosphorylated TrkAIII levels in the TrkAIII SH-SY5Y cells treated for 6 h with DS16570511 plus Dox were significantly enhanced (*p* = 0.044, df = 2) (Figure 3c). Furthermore, Dox significantly augmented the TrkAIII phosphorylation in non-selected TrkAIII SH-SY5Y cells pre-treated for 24 h with DS16570511 but not in the M-phase-enriched, DS16570511-treated counterparts (*p* = 0.044, df = 2). This confirms that Dox-induced TrkAIII activation is Ca^2+^-uniporter-independent and occurs in senescent cells, which are presumably arrested in G1/S but not mitosis [12,37].

The indirect IF also detected reduced TrkAIII phosphorylation in TrkAIII SH-SY5Y cells treated with DS16570511 alone but not cells treated for 6 h with DS16570511 plus Dox (Figure 1b).

The potential c-Src involvement in Dox-induced TrkAIII activation was assessed using the Src inhibitor PP1 [38]. PP1 did not prevent Dox-induced TrkAIII phosphorylation (Figure 3c). A requirement for oxidation for Dox-induced TrkAIII activation was assessed using resveratrol. Resveratrol almost completely abrogated the Dox-induced TrkAIII phosphorylation (*p* = 0.021, df = 2) (Figure 3c). The prevention of both the Dox-induced and constitutive forms of TrkAIII phosphorylation by resveratrol was also detected using indirect IF in TrkAIII SH-SY5Y cells (Figure 1b).

The oxygen requirement for TrkAIII phosphorylation was confirmed under conditions of hypoxia. The hypoxia completely blocked the Dox-induced and constitutive cell cycle-regulated TrkAIII phosphorylation in TrkAIII SH-SY5Y cells, as detected via Western blot and indirect IF (Figure 1b and Figure 3c).

These observations confirm that Dox-induced TrkAIII activation depends upon oxygen and oxidation but not c-Src.

### 2.2. Dox Induces Unconventional Xbp1 Splicing but Does Not Promote Alternative TrkAIII Splicing, in Contrast to Hypoxia

**Figure 4:**
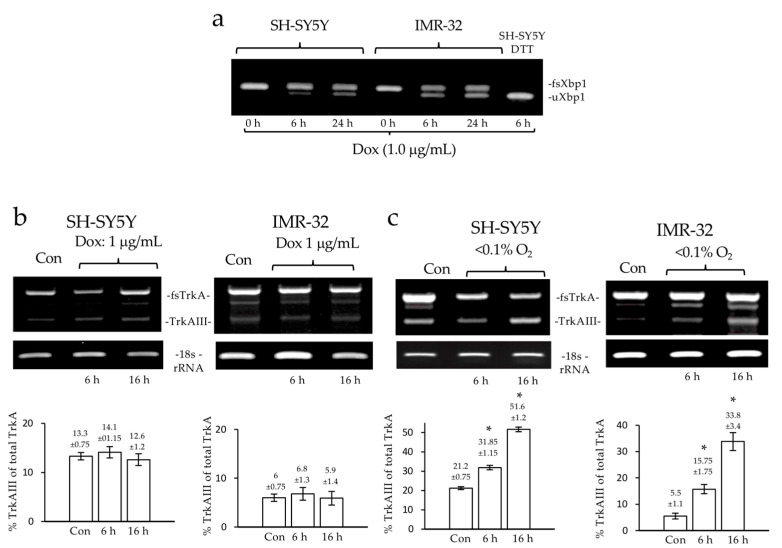
*Dox induces unconventional Xbp-1 splicing in NB cells.* (**a**) Agarose gel, demonstrating the time-dependent induction of unconventional Xbp-1 splicing (uXbp1) in RT-PCRs of RNA samples from SH-SY5Y and IMR-32 NB cells, compared to untreated SH-SY5Y and IMR32 negative controls and DTT (10 mM)-treated SH-SY5Y positive controls, at the concentrations and times indicated. (**b**) Agarose gels and histograms, demonstrating no significant changes in fully spliced TrkA (fsTrkA) and alternatively spliced TrkAIII RT-PCR product levels in RNA samples from SH-SY5Y and IMR32 cells treated with Dox at the concentrations and times indicated. (**c**) Agarose gels and histograms, demonstrating a significant increase (*) in mean (±se) TrkAIII relative to the fully spliced TrkA (fsTrkA) RT-PCR product levels in SH-SY5Y and IMR32 cells treated with hypoxia for the times indicated compared to normoxic controls (Con).

A RT-PCR analysis of unconventional Xbp-1 splicing as an index of ER stress was performed in non-Myc-amplified SH-SY5Y and N-Myc-amplified IMR32 NB cells. The Dox induced unconventional Xbp-1 splicing in both cell lines compared to untreated controls and DTT-treated (10 mM for 6 h) SH-SY5Y cells, as a positive control for unconventional Xbp-1 splicing (Figure 4a).

The RT-PCR analysis of the alternative TrkAIII splicing revealed that Dox did not promote alternative TrkAIII mRNA splicing in SH-SY5Y or IMR32 cells (Figure 4b).

In contrast to Dox, the RT-PCRs confirmed that hypoxia promotes alternative TrkAIII mRNA splicing in SH-SY5Y (*p* = 0.0173 at 6 h and *p* = 0.002 at 12 h, df = 2) and IMR32 cells (*p* = 0.038 at 6 h and *p* = 0.016 at 24 h, df = 2) (Figure 4c).

### 2.3. Dox-Induced TrkAIII Activation Involves Ryanodine Receptors (RyRs), Calmodulin (CaM), Hsp90, and ARF-1-Dependent ERGIC Assembly

**Figure 5:**
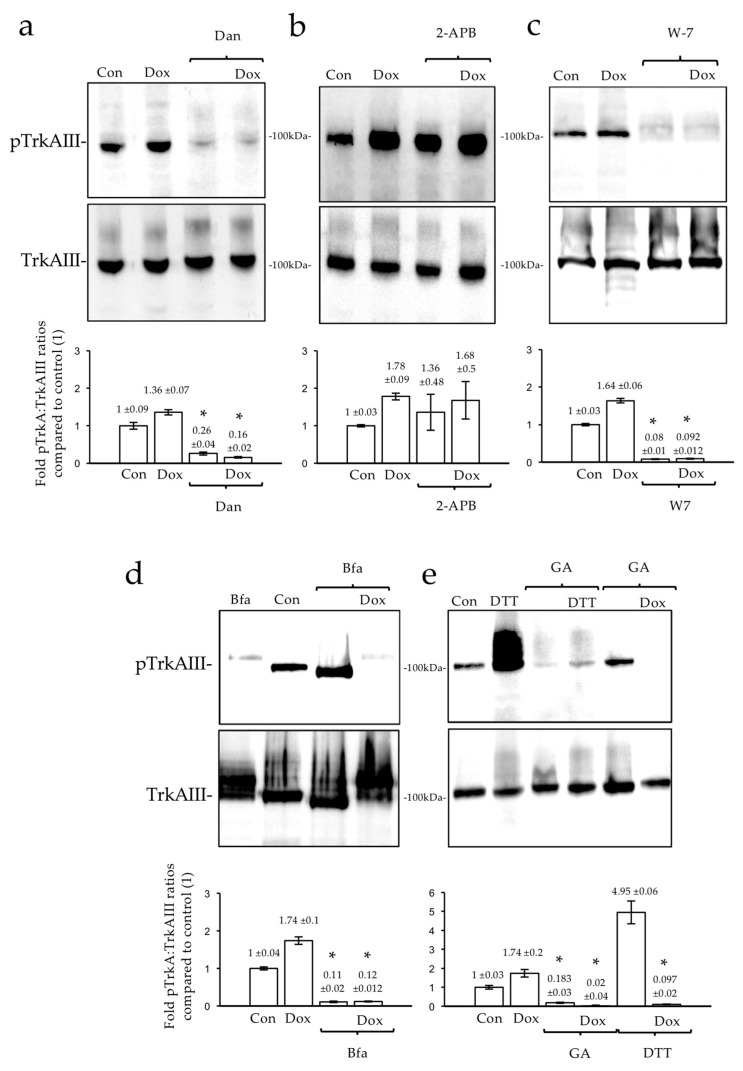
*Dox-induced TrkAIII activation is RyR-, CaM-, and ARF-1-dependent.* Western blots and histograms, demonstrating the following: (**a**) significant (*) reductions in mean (±se) cell-cycle-regulated (Con) and Dox-induced (Dox) TrkAIII phosphorylation, relative to the total TrkAIII levels in TrkAIII SH-SY5Y cells treated for 6 h with dantrolene (10 μM) alone (Dan) or dantrolene (10 μM) plus Dox (1 μg/mL) (Dan/Dox); (**b**) no reductions in either constitutive or Dox (1 μg/mL)-induced TrkAIII phosphorylation in TrkAIII SH-SY5Y cells treated with the IP3R inhibitor 2-APB (100 μM); (**c**) the abrogation of both constitutive (Con) and Dox (1 μg/mL for 6 h)-induced forms of TrkAIII phosphorylation in TrkAIII SH-SY5Y cells treated with W7 (60 μM); (**d**) the abrogation of constitutive (Con) and Dox (1 μg/mL for 6 h)-induced TrkAIII phosphorylation in TrkAIII SH-SY5Y cells treated with brefeldin A (5 μg/mL) (Bfa), (**e**) the significant inhibition of constitutive, DTT (10 mM for 6 h)-induced, and Dox (1 μg/mL for 6 h)-induced TrkAIII phosphorylation in TrkAIII SH-SY5Y cells treated with geldanamycin (1 μM) (GA).

To investigate the potential involvement of stress-regulated ER calcium channels in TrkAIII activation, the TrkAIII SH-SY5Y cells were treated with the RyR inhibitor dantrolene [39] and the IP3R inhibitor 2-APB [40]. Dantrolene but not 2-APB, at the concentrations and time indicated, significantly reduced both the Dox-induced and constitutive cell cycle-regulated forms of TrkAIII phosphorylation in TrkAIII SH-SY5Y cells (*p* = 0.0037 and *p* = 0.0229, respectively, df = 2) (Figure 5a,b).

To assess the involvement of Ca^2+^-dependent CaM in TrkAIII phosphorylation, the TrkAIII SH-SY5Y cells were treated with the Ca^2+^-dependent CaM inhibitor W-7 [41]. W7, at the concentration and time indicated, abrogated both the Dox-induced and constitutive cell cycle-regulated forms of TrkAIII phosphorylation in TrkAIII SH-SY5Y cells (*p* = 0.0012, and *p* = 0.0092, respectively df = 2) (Figure 5c).

To determine whether the Dox-induced TrkAIII activation impacts upon the ARF-1-dependent ERGIC assembly, the TrkAIII SH-SY5Y cells were treated with the ERGIC-dissociating ARF-1 inhibitor, brefeldin A (Bfa) [42]. Bfa, at the concentration and time indicated, significantly reduced both the Dox-induced and constitutive cell cycle-regulated forms of TrkAIII phosphorylation in TrkAIII SH-SY5Y cells (*p* = 0.0143 and *p* = 0.02, respectively df = 2) (Figure 5d).

To assess Hsp90’s involvement in Dox-induced TrkAIII phosphorylation, TrkAIII SH-SY5Y cells were treated with the HSP90 inhibitor geldanamycin (GA) [43]. GA, at the concentration and time indicated, significantly reduced the Dox-induced, DTT-induced, and constitutive cell cycle-regulated TrkAIII phosphorylation in TrkAIII SH-SY5Y cells (*p* = 0.0027, *p* = 0.0024 and *p* = 0.0007, respectively, df = 2) (Figure 5e).

Together, these observations indicate that the Dox-induced and constitutive cell cycle-regulated TrkAIII forms of activation depend upon the RyRs, CaM, Hsp90, and ARF-dependent ERGIC assembly.

### 2.4. TrkAIII Complexes with CaM

**Figure 6:**
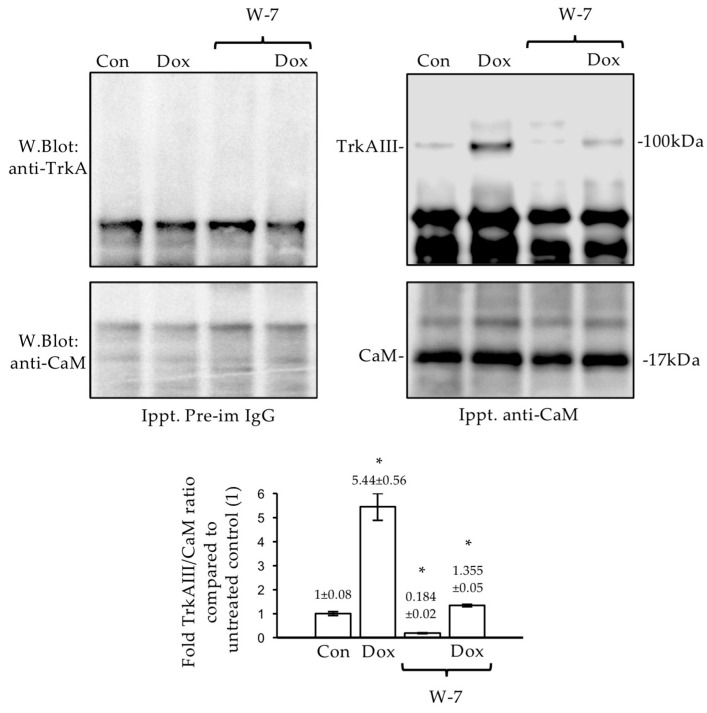
*Dox enhances CaM/TrkAIII complexing.* Pre-immune IgG and anti-CaM co-immunoprecipitation Western blots and histograms, demonstrating a significant increase (*) in constitutive CaM pull-down of TrkAIII in untreated TrkAIII SH-SY5Y cells (Con), following treatment with Dox (1 μg/mL for 6 h), and significant reductions (*) in both constitutive and Dox-enhanced CaM/TrkAIII pull-down in cells treated with W-7 (60 μM) (Dox/W-7).

To further investigate CaM’s involvement in the TrkAIII phosphorylation, co-immunoprecipitation assays were performed. The co-immunoprecipitation Western blotting revealed that the constitutive CaM pull-down of TrkAIII detected in extracts from untreated TrkAIII SH-SY5Y cell extracts was significantly augmented by the Dox treatment (*p* = 0.0158 df = 2). Treatment with W7, at the concentration and time indicated, reduced both the constitutive and Dox-enhanced forms of CaM/TrkAIII complexing (*p* = 0.0097 and *p* = 0.0182, respectively df = 2) (Figure 6). This implicates Ca^2+^-dependent CaM/TrkAIII complexing in Dox-induced TrkAIII phosphorylation.

### 2.5. Dox-Induced TrkAIII Activation Enhances Akt Phosphorylation in TrkAIII SH-SY5Y Cells

**Figure 7:**
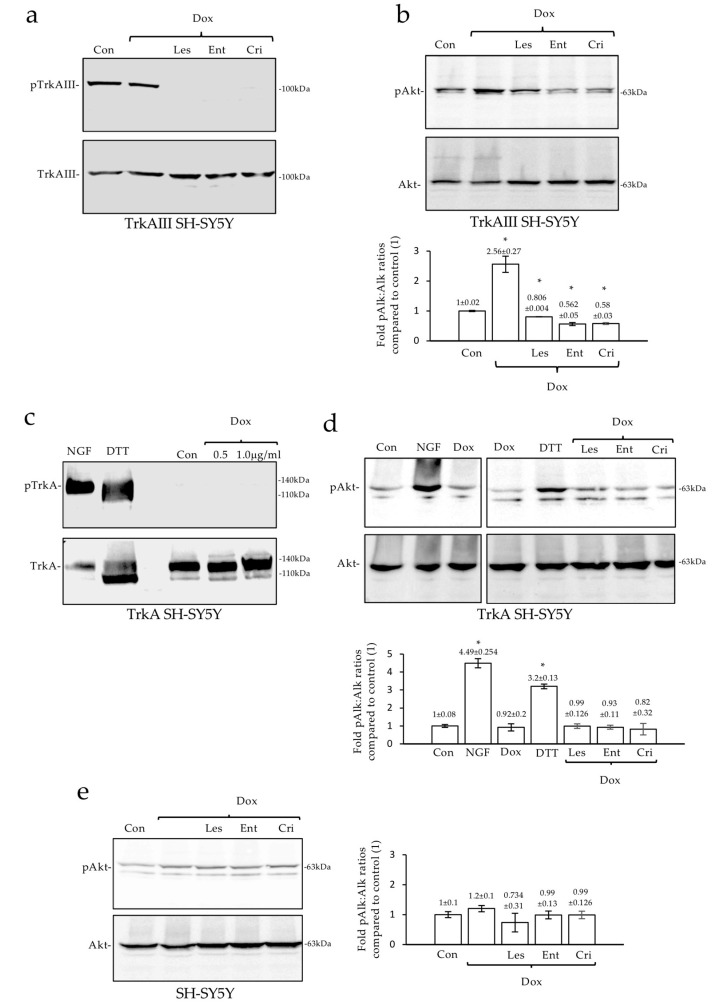
*Dox induces TrkAIII and Akt phosphorylation but not TrkA phosphorylation.* (**a**) Western blots demonstrating complete inhibition of TrkAIII phosphorylation induced by 6 h treatment with Dox (1 μg/mL) alone, and in combination with lestaurtinib (1 μM) (Les/Dox), entrectinib (1 μM) (Ent/Dox), and crizotinib (100 nM) (Cri/Dox). (**b**) Western blots and a histogram, demonstrating significant changes (*) in Akt phosphorylation in untreated TrkAIII SH-SY5Y cells (Con), following 6 h treatments with Dox (1 μg/mL) alone and in combination with lestaurtinib (1 μM) (Les/Dox), entrectinib (1 μM) (Ent/Dox), and crizotinib (100nM) (Cri/Dox). (**c**) Western blots demonstrating the induction of TrkA phosphorylation in TrkA SH-SY5Y cells by NGF (100 ng/mL for 15 min) and DTT (10 mM for 6 h) but not by Dox at the concentrations indicated for 6 h. (**d**) Western blots and a histogram, demonstrating significant increases (*) in Akt phosphorylation in NGF and DTT but not Dox-treated TrkA SH-SY5Y cells, at the above concentrations and times, and no changes in Akt phosphorylation in TrkA SH-SY5Y cells treated for 6 h with Dox (1 μg/mL) plus lestaurtinib (1 μM) (Les/Dox), entrectinib (1 μM) (Ent/Dox), or crizotinib (100 nM) (Cri/Dox). (**e**) Western blots and a histogram, demonstrating no differences in Akt phosphorylation in untreated parental SH-SY5Y cells (Con) and cells treated for 6 h with Dox (1 μg/mL) alone or in combination with lestaurtinib (1 μM) (Les/Dox), entrectinib (1 μM) (Ent/Dox), or crizotinib (100 nM) (Cri/Dox).

To determine the relationship between Dox-induced TrkAIII activation and Akt phosphorylation, the TrkAIII and Akt forms of phosphorylation were compared in untreated and Dox-treated TrkAIII SH-SY5Y cells, in the presence and absence of lestaurtinib [44], entrectinib [45], and crizotinib [46] via Western blot. Lestaurtinib, entrectinib, and crizotinib, at the concentrations and times indicated, all abrogated the Dox-induced TrkAIII phosphorylation (Figure 7a). The Dox significantly increased the Akt phosphorylated in TrkAIII SH-SY5Y cells (*p* = 0.0288, df = 2), which was significantly reduced by lestaurtinib, entrectinib, and crizotinib (*p* = 0.0229, *p* = 0.562 and *p* = 0.0183, respectively, df = 2) (Figure 7b). This implicates TrkAIII in Dox-induced Akt phosphorylation.

### 2.6. Dox Does Not Activate Fully Spliced TrkA nor Enhance Akt Phosphorylation in TrkA SH-SY5Y Cells

To determine whether Dox-induced activation is specific for TrkAIII, the effect of Dox was also assessed in TrkA SH-SY5Y cells, which express fully spliced inactive cell surface gp140 TrkA [26,45]. The Western blots revealed that NGF and DTT but not Dox induce TrkA phosphorylation in TrkA SH-SY5Y cells (Figure 7c). The constitutive Akt phosphorylation in TrkA SH-SY5Y cells was significantly enhanced by NGF and DTT (*p* = 0.0058 and *p* = 0.0095, respectively, df = 2) but not by Dox, and was not altered by lestaurtinib, entrectinib, or crizotinib (Figure 7d). The constitutive Akt phosphorylation in parental SH-SY5Y cells was also not altered by Dox, lestaurtinib, entrectinib, or crizotinib (Figure 7e). These observations further confirm TrkAIII’s involvement in Dox-enhanced Akt phosphorylation.

### 2.7. Dox-Induced TrkAIII Activation Enhances Resistance to Dox Cytotoxicity and Is Reversed by Trk and IP3-K Inhibitors

**Figure 8:**
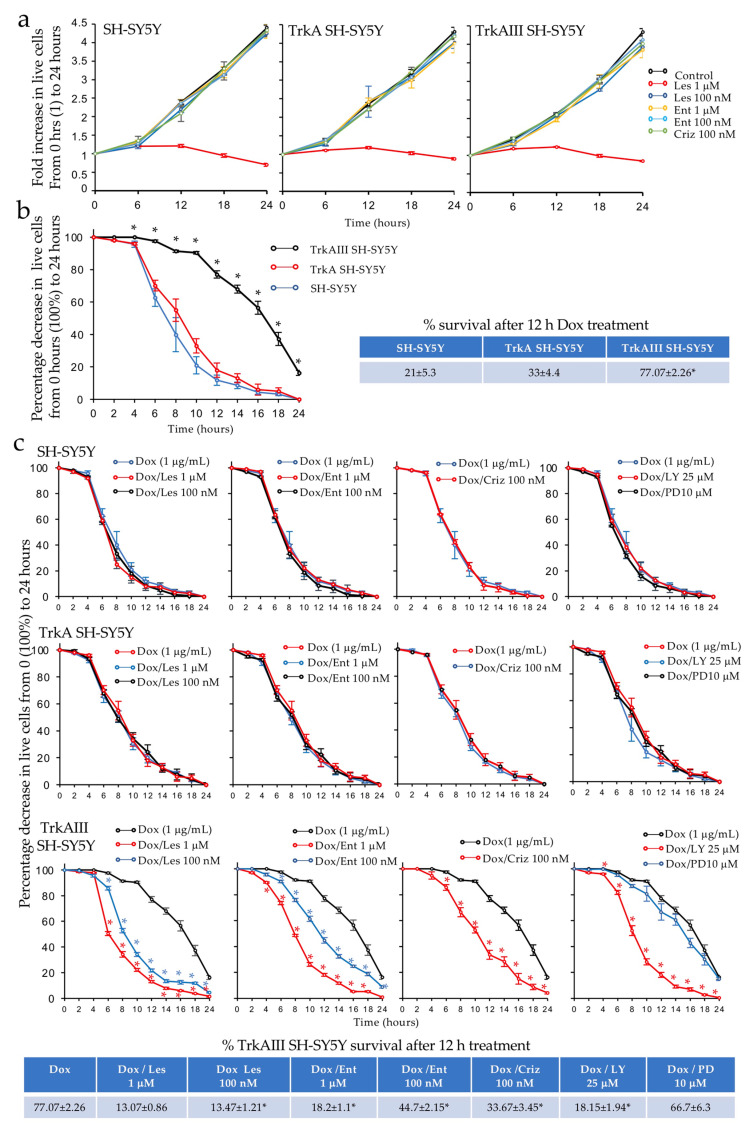
*Dox-induced TrkAIII activation confers Dox resistance to SH-SY5Y cells.* (**a**) Line graphs demonstrating SH-SY5Y, TrkA SH-SY5Y, and TrkAIII SH-SY5Y proliferation rates under untreated conditions and in the presence of lestaurtinib (Les), entrectinib (Ent), and crizotinib (Criz), at the concentrations indicated, over a 24 h time course. (**b**) Line graphs demonstrating significantly enhanced (*) TrkAIII SH-SY5Y survival compared to SH-SY5Y and TrkA SH-SY5Y survival in the presence of 1 μg/mL Dox, over a 24 h time course (* = statistical significance), plus a table reporting the mean (±se) percentage survival rates of SH-SY5Y, TrkA SH-SY5Y, and TrkAIII SH-SY5Y cells following Dox treatment at 12 h. (**c**) Line graphs demonstrating significant (*) reductions in TrkAIII SH-SY5Y survival in the presence of Dox (1 μg/mL) combined with lestaurtinib (Les), entrectinib (Ent), crizotinib, (Criz), and LY294002 (LY) but not PD08059 (PD) at the concentrations indicated, compared to SH-SY5Y and TrkA SH-SY5Y survival under the same conditions over a 24 h time course (* = statistical significance), plus a table reporting the mean (±se) percentage survival of TrkAIII SH-SY5Y cells following 12 h treatment with Dox alone and in combination with lestaurtinib (Les), entrectinib (Ent), crizotinib, (Criz), LY294002 (LY), and PD08059 (PD) at the concentrations indicated In IncuCyte assays. Dox was significantly more cytotoxic to SH-SY5Y and TrkA SH-SY5Y cells than TrkAIII SH-SY5Y cells, which exhibited significantly enhanced survival at all times from 2 to 24 h when compared to both SH-SY5Y and TrkA SH-SY5Y cells (Figure 8b). Survival rates in the presence of Dox did not significantly differ in parental SH-SY5Y and TrkA SH-SY5Y cells over 24 h. To illustrate this, compared to 0 h cultures (100% live cells), Dox-treatment for 12 h reduced the parental SH-SY5Y survival rate to 21%, reduced the TrkA SH-SY5Y cell survival rate to 33%, but only reduced the TrkAIII SH-SY5Y cell survival rate to 77% (*p* < 0.0001 TrkAIII SH-SY5Y verses both SH-SY5Y and TrkA SH-SY5Y cells, df = 10) (Figure 8b).

To investigate the biological effects of Dox-induced TrkAIII activation, the growth and survival of SH-SY5Y, TrkASH-SY5Y, and TrkAIII SH-SY5Y cells were compared under different treatment conditions by live cell IncuCyte assay. In untreated conditions, the SH-SY5Y, TrkA SH-SY5Y, and TrkAIII SH-SY5Y cells did not exhibit differences in proliferation rates and exhibited similar doubling times of ≈12 h (Figure 8a). This confirms that inactive TrkA and constitutively active TrkAIII do not influence SH-SY5Y proliferation.

In inhibitor studies, entrectinib and crizotinib, at the concentrations and time indicated, did not significantly inhibit SH-SY5Y, TrkA SH-SY5Y, or TrkAIII SH-SY5Y proliferation over 24 h (Figure 8a). In contrast, lestaurtinib at 1 μM but not at 100 nM almost completely abrogated the proliferation of all 3 cell lines (*p* < 0.0001, for all 3 cell lines at all time points). This confirms that TrkAIII does not regulate SH-SY5Y proliferation and suggests that the proliferation depends upon a lestaurtinib-sensitive tyrosine kinase.

**Figure 9:**
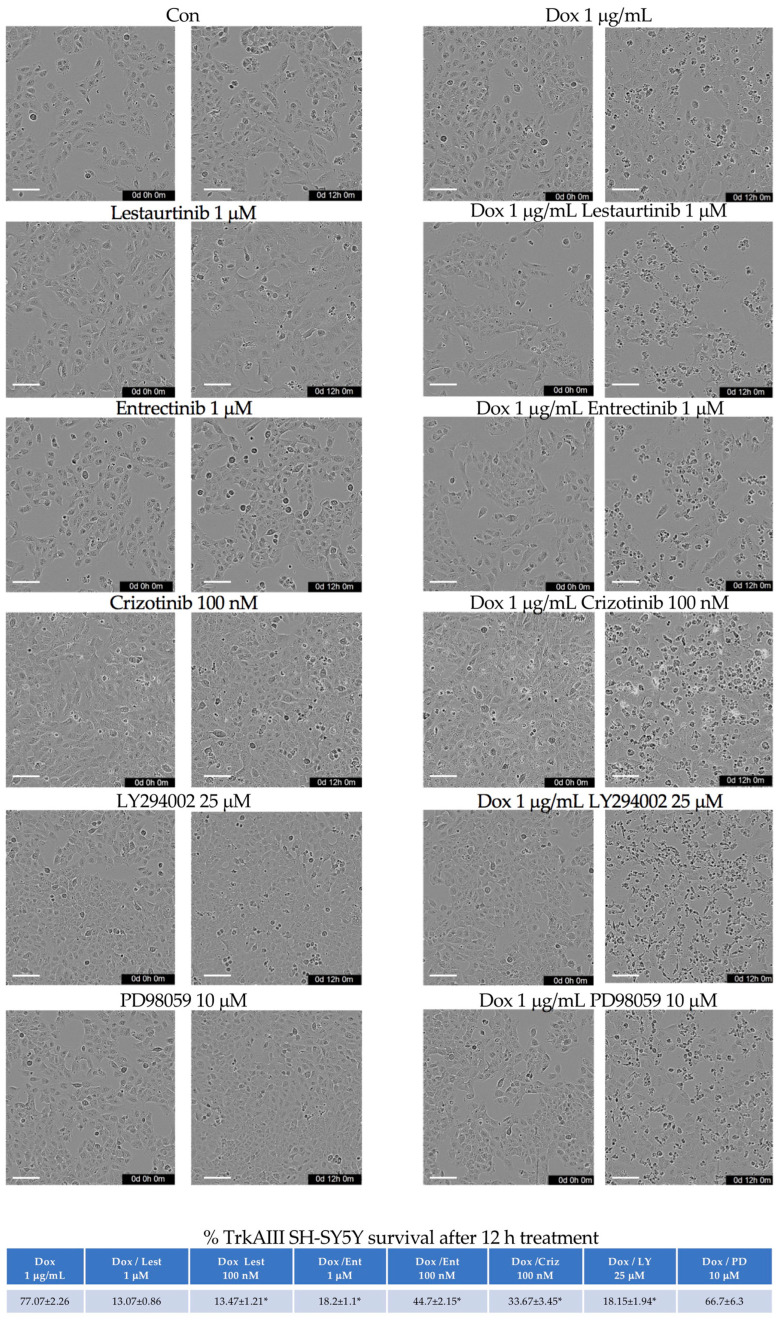
*Trk inhibitors enhance Dox-induced TrkAIII SH-SY5Y cytotoxicity.* IncuCyte phase contrast micrographs, demonstrating changes in live and dead or dying cell levels in untreated TrkAIII SH-SY5Y cells and cells treated for 0 and 12 h with lestaurtinib, entrectinib, crizotinib, LY294002, and PD98059 alone and in combination with Dox at the concentrations listed (bar = 100 μm, * = statistical significance).

To determine the roles of TrkAIII, IP3-K, and MEK/MAPKs in Dox resistance, the survival of SH-SY5Y, TrkA SH-SY5Y, and TrkAIII SH-SY5Y was evaluated following treatment with Dox alone and in combination with lestaurtinib, entrectinib, and crizotinib, as well as the IP3-K inhibitor LY294002 [47] and the MEK/MAPK inhibitor PD098059 [48], at the concentrations and time indicated. The survival of the Dox-treated SH-SY5Y and TrkA SH-SY5Y cells was not significantly altered by lestaurtinib, entrectinib, crizotinib, LY294002, or PD98059 (Figure 8c). In contrast, the survival of the Dox-treated TrkAIII SH-SY5Y cells was significantly reduced by lestaurtinib, entrectinib, crizotinib, and LY294002 but not by PD98059 (Figure 8c). To illustrate this, the survival of TrkAIII SH-SY5Y following the Dox treatment for 12 h was significantly reduced to ≈13% by lestaurtinib at both concentrations (*p* < 0.0001, df = 8), to ≈18% by 1 μM and ≈45% by 100 nM entrectinib (*p* < 0.0001, df = 8), to ≈34% by crizotinib (*p* < 0.0001, df = 8), and to ≈18% by LY294002, but was not significantly reduced by PD98059 (Figure 8c). The effects of the tyrosine kinase inhibitors and LY294002 on TrkAIII SH-SY5Y survival in the absence and presence of Dox are also demonstrated in the IncuCyte micrographs presented in Figure 9.

### 2.8. SH-SY5Y, TrkA SH-SY5Y, and TrkAIII SH-SY5Y Cells Do Not Carry the NM_004304.5 (ALK):c.3522C > A (p.Phe1174Leu) Mutation

**Figure 10:**
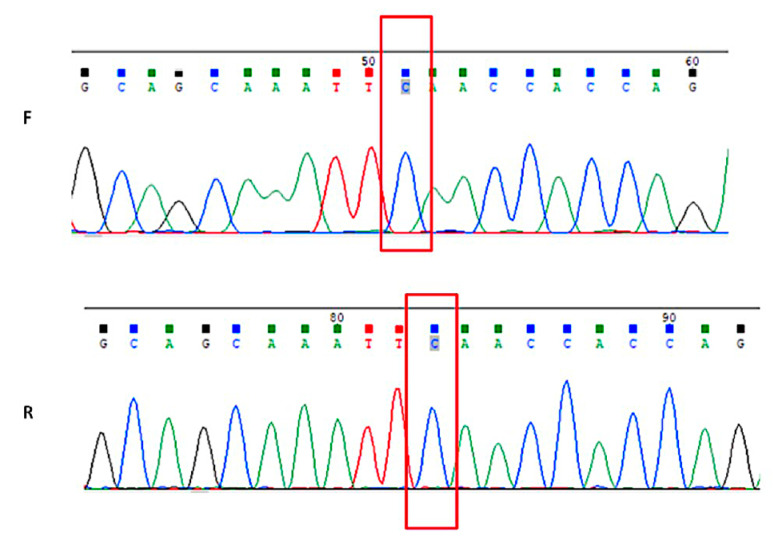
*SH-SY5Y cells do not carry theNM_004304.5 (ALK):c.3522C > A (p. Phe1174Leu) mutation*. Genomic c.3522C > A Alk mutation site sequence, demonstrating the absence of the NM_004304.5(ALK):c.3522C > A (p. Phe1174Leu) mutation in parental SH-SY5Y cells.

The direct PCR sequencing of genomic DNA using primers spanning the NM_004304.5 (ALK):c.3522C > A (p.Phe1174Leu) mutation, as previously reported in SH-SY5Y cells [49,50], did not detect this mutation in either parental SH-SY5Y, TrkA SH-SY5Y, or TrkAIII SH-SY5Y cell lines (Figure 10, displayed for SH-SY5Y cells only). This explains why the ALK inhibitors entrectinib and crizotinib did not inhibit the proliferation of our SH-SY5Y cells. This cell line was originally obtained from the NIH (Bethesda MD, USA) in 1986, 3 years after its original publication [51], suggesting that the recently described ALK mutation in SH-SY5Y cells [49,50] may have been acquired in culture [52].

## 3. Discussion

We report a novel Dox resistance mechanism in human NB cells characterized by Dox-induced activation of the oncogenic alternative TrkAIII splice variant. This mechanism is specific for TrkAIII, does not affect fully spliced TrkA, occurs within assembled ERGICs of cells arrested in proliferation, and enhances Dox resistance though IP3-K/Akt signaling. The Dox-induced TrkAIII activation depends upon RyRs, Ca^2+^/calmodulin, and HSP90; requires oxygen and oxidation; and is associated with UPR activation. It differs from cell-cycle-regulated TrkAIII activation in that it is independent of CDK1 and the Ca^2+^ uniporter, and is abrogated by lestaurtinib, entrectinib, and crizotinib tyrosine kinase and LY294002 IP3-K inhibitors.

The specificity of this mechanism for TrkAIII reflects the omission of the extracellular D4 domain encoded within skipped exons 6 and 7. This domain is required for the rapid cell surface translocation of fully spliced TrkA, confines intracellular TrkA to the Golgi compartment, and prevents spontaneous TrkA activation [27,28,29,30,31,43]. The D4 domain deletion in TrkAIII abolishes cell surface expression, promotes intracellular pre-Golgi accumulation, and facilitates spontaneous activation [27,28]. Although severe ER stress prevents TrkA maturation and promotes immature TrkA intracellular accumulation and spontaneous activation, as shown in this study and [53], the level of ER stress induced by Dox, as confirmed by unconventional Xbp-1 splicing, did not prevent TrkA maturation nor promote intracellular TrkA accumulation and activation, further explaining the specificity of this mechanism for TrkAIII.

Dox’s activation of TrkAIII in senescent TrkAIII SH-SY5Y cells arrested in proliferation by Dox-induced DNA damage and DNA damage response activation [12,37] and by the CDK1 inhibitor RO-3306 [36] confirms a CDK1- and cell-cycle-independent activation mechanism. In contrast, RO-3306’s inhibition of constitutive TrkAIII phosphorylation in the absence of Dox confirms that the TrkAIII activation in proliferating cells is CDK1-dependent and cell-cycle-regulated. The constitutive cell-cycle-regulated TrkAIII phosphorylation in TrkAIII SH-SY5Y cells is initiated following cytokinesis, decreases as the cell cycle progresses, and is inhibited during mitosis, as shown in this study and [54]. Therefore, Dox-induced TrkAIII phosphorylation in senescent proliferation-inhibited TrkAIII SH-SY5Y cells may result from arrest in an activation-permissive post-division state or caused by the substitution of TrkAIII phosphorylation inhibitory conditions associated with cell cycle progression, and is confined to a non-mitotic state characterized by ERGIC assembly.

DS16570511’s inhibition of cell-cycle-regulated but not Dox-induced TrkAIII activation confirms the dependence upon the mitochondrial Ca^2+^ uniporter for the former but not the latter activation mechanism. For cell-cycle-regulated TrkAIII activation, this can be partly explained by the DS16570511-induced M-phase arrest [35], as characterized by ERGIC dissociation and the inhibition of TrkAIII phosphorylation. Furthermore, DS16570511 inhibits the Ca^2+^ uniporter-mediated metabolic burst required for M-phase completion and cytokinesis [35], intrinsically linking the Ca^2+^ uniporter to TrkAIII phosphorylation post-cytokinesis. The suggests that this metabolic burst also provides the oxidative conditions for transient TrkAIII activation post-cytokinesis, as supported by hypoxia and resveratrol’s prevention of cell-cycle-regulated TrkAIII activation. DS16570511’s inability to prevent Dox-induced TrkAIII activation, which substitutes the reliance upon the Ca^2+^ uniporter, can be explained by Dox’s generation of ROS at multiple intracellular sites via interactions with NADPH/NADP and oxygen [55]. This would provide the oxidative conditions necessary for intracellular TrkAIII activation through PTPase inhibition [9], and is also sustained by hypoxia and resveratrol’s prevention of Dox-induced TrkAIII activation, confirming the dependence upon oxygen and oxidation.

The prevention of Dox-induced TrkAIII activation by dantrolene [39] but not by 2-APB [40] implicates RyRs but not IP3Rs in this activation mechanism. This is supported by reports that Dox binds and activate RyRs, including RyR2, which is expressed by SH-SY5Y cells [56,57,58,59]. Dantrolene also blocked cell-cycle-regulated TrkAIII activation, which may be explained by the activation of an UPR adaptation in TrkAIII in SH-SY5Y cells [43], as the UPR activation promotes RyR-mediated ER Ca^2+^ release [60].

Ca^2+^ and CaM’s involvement in Dox-induced TrkAIII activation was confirmed by W-7’s [41] prevention of Dox-induced TrkAIII activation and Dox-enhanced TrkAIII/CaM complexing. This confirms and extends a report that CaM binds fully spliced TrkA [61] to include TrkAIII and suggests that Dox-induced TrkAIII activation depends upon RyR-mediated ER-Ca^2+^ release and Ca^2+^-dependent CaM/TrkAIII complexing. CaM also regulates the activity of Hsp90 [62], and the Hsp90 inhibitor GA prevented Dox-induced TrkAIII activation. This confirms a previous report that TrkAIII activation is Hsp90-dependent [43] and suggests that Dox-induced TrkAIII activation involves complex-Ca^2+^-regulated interactions between TrkAIII, CaM, and Hsp90, which may also extend to cell-cycle-regulated TrkAIII activation.

The significantly enhanced resistance to Dox cytotoxicity exhibited by TrkAIII SH-SY5Y NB cells was abrogated by lestaurtinib, entrectinib, and crizotinib at TrkAIII inhibitory concentrations and by the IP3K inhibitor LY294002 but not by the MEK/MAPK inhibitor PD98059. This implicates TrkAIII and IP3-K but not MEK/MAPKs in this Dox resistance mechanism. Tyrosine kinase and IP3K but not MEK/MAPK inhibitors also prevented the increase in Akt phosphorylation induced by Dox in TrkAIII SH-SY5Y. This suggests that this mechanism results from Dox-induced, TrkAIII-mediated, IP3-K/Akt-dependent pro-survival signaling. This is consistent with previous reports that TrkAIII induces pro-survival IP3-K/Akt signaling [27,28] and that Akt mediates Dox resistance in NB cells [18,19,20,21,22,23]. The induction of unconventional Xbp-1 splicing by Dox negates the inhibition of the Ire1a/XBP1 arm of the UPR in this resistance mechanism [63], while PP1 did not prevent Dox-induced TrkAIII activation, indicating that any potential Src involvement would be downstream of TrkAIII [24]. As TrkAIII promotes a stem-cell-like NB phenotype [64], we do not exclude potential roles for stem-cell- or EMT-associated Dox resistance gene expression [13,17] in this mechanism.

The dependence of the Dox-induced TrkAIII activation upon oxygen, combined with the observation that Dox does not promote alternative TrkAIII mRNA splicing, despite activating the UPR, indicates that this TrkAIII-mediated Dox resistance mechanism would be confined to oxygenated TMEs populated by tumor cells that already express TrkAIII. Alternative TrkAIII splicing in NB cells is promoted by agents that induce severe ER stress, by the SV40 polyomavirus large T antigen, and by the hypoxia mimic CoCl_2_ [27,28], providing three conditions though which TrkAIII expression may be promoted in NBs. Here, we confirm and extend these reports to show that bona fide conditions of hypoxia promote alternative TrkAIII splicing but prevent Dox-induced TrkAIII activation in NB cells. This suggests that cyclic hypoxia and reoxygenation, which characterize 20–30% of TMEs at any particular time [65], may provide ideal conditions for activating this Dox resistance mechanism. TrkAIII expression promoted by acute transient hypoxia would be followed by reoxygenation-dependent TrkAIII activation upon blood flow restoration and the arrival of Dox. This, in association with Dox-induced proliferation arrest and senescence [12], would provide a mechanism to select resistant, dormant TrkAIII-expressing NB cells, increasing the potential for post-therapeutic relapse and metastatic progression [12,27,28].

In conclusion, we propose that Dox-induced TrkAIII activation in NB cells resulting in pro-survival IP3-K/Akt signaling enhances the resistance to Dox cytotoxicity, and is inhibited by clinically approved lestaurtinib, entrectinib, and crizotinib inhibitors. The potential for this novel Dox resistance mechanism to promote post-therapeutic relapse and metastatic progression in NBs expressing TrkAIII provides a rational for including approved Trk inhibitors, such as lestaurtinib, entrectinib, and crizotinib, during induction-phase chemotherapy with Dox.

## 4. Materials and Methods

### 4.1. Cell Lines and Culture Conditions

Stable transfected TrkA and TrkAIII SH-SY5Y lines were generated from parental SH-SY5Y NB cells were provided by DR. U.P. Thorgeirsson (NCI, NIH Bethesda, MD, USA), and have been previously described [26]. The IMR32 NB cell line (ATCC CCL-127) was from the ATCC (Manassas, VI, USA). The NB cell lines were routinely cultured in RPMI1640, supplemented with 10% fetal bovine serum (Euroclone, Milan, Italy), 1% penicillin/streptomycin, and Zeocin for stable transfectants (ThermoFisher Scientific, Waltham, MA, USA), and 1% glutamine (Euroclone, Milan, Italy) at 37 °C and 5% CO_2_. For conditions of hypoxia, the cells were cultured in conditions of <0.1 % O_2_ and 5% CO_2_, in RPMI1640 medium without glucose (Euroclone, Milan, Italy), and were previously degassed for 24 h in a New Brunswick Galaxy 48R hypoxia incubator (Eppendorf Company, Hamburg, Germany). The cell growth, survival, and death were evaluated via time lapse digital photography at 10× magnification in an IncuCyte^®^ S3 Live-Cell Analysis System (Sartorius, Goettingen, Germany).

#### Reagents and Antibodies

The doxorubicin hydrochloride, dithiothreitol (DTT), A23187 calcium ionophore, Geldanamycin, Brefeldin A, Dantrolene, 2-aminoethyl diphenylborinate (2-APB), PP1 Src inhibitor, PD98059 MEK/MAPK inhibitor, LY294002 IP3-K inhibitor, the pan Trk inhibitor lestaurtinib, NGF, and Protein A Sepharose (Fast Flow) were from Sigma-Aldrich (Saint Louis, MO, USA). The crizotinib and RO-3306 were from Med Chem Express (Monmouth Junction, NJ, USA). The entrectinib was from Selleck Chemicals (Houston, TX, USA), DS16570511 was from Cayman Chemical (Ann Arbour MI, USA), the resveratrol was from ENZO Life Sciences Inc (Farmingdale, NY, USA), and the W-7 was from Millipore Sigma (Burlington, MA, USA). The polyclonal rabbit anti-human-TrkA (B3, sc-7268, 200 μg/mL) and mouse monoclonal anti-human calmodulin (sc-137079, 200 μg/mL) antibodies were from SantaCruz (Santa Cruz, CA, USA). The rabbit polyclonal anti-human Y490 phosphorylated TrkA (9141, 36 μg/mL), polyclonal rabbit anti-human Akt (9272, 31 μg/mL), and polyclonal rabbit anti-human phospho-Ser 473-Akt (4060, 91 μg/mL) antibodies were from Cell Signaling Technology (Danvers, MA, USA). The secondary antibodies, which included horseradish peroxidase (HRP)-conjugated goat anti-rabbit and rabbit anti-mouse antibodies (1 mg/mL), were from Bethyl Laboratories Inc. (Fortis, Waltham, MA, USA), while the Alexa Fluor 488-labeled donkey anti-rabbit and Alexa Fluor donkey anti-mouse antibodies were from Life Technologies (1 mg/mL) (Waltham, MA, USA). The ProLong^TM^ Gold anti-fade reagent with DAPI was from Invitrogen (Thermo-Fisher Scientific, Waltham, MA, USA).

### 4.2. Two-Dimensional Growth, Cell Death, and Growth Assays

The two-dimensional growth was assessed over a 24 h time course, in the presence or absence of different inhibitors, in an IncuCyte^®^ S3 Live-Cell Analysis System incubator, as directed (Sartorius, Goettingen, Germany). Briefly, the cells were seeded at a density of 1 × 10^3^ per well in 96-well cell culture plates (353072, Corning Inc, NY, USA), allowed to attach for 4 h at 37 °C, and then treated with inhibitors at different concentrations, as indicated in the figures and figure legends. IncuCyte analysis software was programmed for time lapse photography of 2 independent areas per well, at 2 h intervals and 10× magnification. The cell behavior was analyzed via time lapse video and cell growth and survival curves constructed by direct cell counting of the phase contrast micrographs. The cells with overt cytoplasmic and nuclear blebbing, or cytoplasmic swelling and necroptotic- or paraptosis-like lysis, were considered to be dead or dying, while the cells exhibiting relatively normal nuclear and cytoplasmic morphologies were considered living. The living and dead or dying cells were counted in 4 independent fields per photograph and the ratio of living to dead or dying cells was calculated. All experiments were performed in duplicate and repeated at least three times.

### 4.3. RNA Purification and RT-PCR

The reverse transcription (RT) reactions were performed on total RNA samples (1 μg) purified using a Quick-RNA™ Miniprep Kit, as described by the manufacturer (Zymo Research, Freiberg im Breisgau, GE), and reverse-transcribed using a Superscript IV reverse transcription kit, as directed (Thermo Fischer Scientific, Waltham, MA, USA). The RT-PCRs were performed using primers sets specific for 18s rRNA (5′-AAACGGCTACCACATCCAAG-3′ and 5′-CCTCGAAAGAGTCCTGTATTG-3′) on 1 μL of RT diluted 1:1000 (0.05 ng) and consisting of 35 cycles of 30 s at 95 °C, 30 s at 58 °C, and 30 s at 72 °C. For the TrkA exons 1–8 and the primers (5′-ATGCTGCGAGGCGGACGGCGC-3′ and 5′-GGAGGCCTGG CCGAAGGGGTT-3′), the RT-PCRs were performed on 1 μL of non-diluted RT (50 ng) and consisted of 35 cycles of 1 min at 95 °C, 30 s at 68 °C, and 1 min at 72 °C. For the detection of unconventional Xbp-1 splicing, RT-PCRs using Xbp-1-specific primers (5′-TTACGAGAGAAAACTCATGGC-3′ and 5′-CGGTCCAAGTTGTCCAGAATGC-3′) were performed on 1 μL of undiluted RT (50 ng) and consisted of 35 cycles of 40 s at 95 °C, 30 s at 59 °C, and 40 s at 72 °C. All RT-PCRs were performed in duplicate and repeated a minimum of twice.

### 4.4. Genomic DNA Extraction and Sequencing

The genomic DNA samples from SH-SY5Y and stable transfected TrkA and TrkAIII SH-SY5Y cell lines were extracted using a Promega Wizard Genomic DNA purification kit, as directed (Promega, Madison, WI, USA). The NM_004304.5 (ALK):c.3522C > A (p.Phe1174Leu) mutation [48,49] was evaluated in an ABI PRISM 310 genetic analyzer (Applied Biosystems, Waltham, MA, USA) via the double-stranded Sanger sequencing of PCR products, which were generated using the Alk-specific primers (5′-TCCTGTTCCTCCCAGTTTAAGA-3′ and 5′-CACTCTTGCTCCTTCCATCC-3′). The PCR products were purified using a EuroSAP PCR Enzymatic Clean-Up Kit as directed (Euroclone, Milan, Italy) and were sequenced using a BigDye Terminator V.2.1. Cycle Sequencing Kit as directed (Thermo-Fisher Scientific, Waltham, MA, USA). The sequencing of genomic ALK DNA in SH-SY5Y, TrkA SH-SY5Y, and TrkAIII SH-SY5Y cells was performed in forward and reverse directions and repeated.

### 4.5. Protein Extraction and Western Blotting

The cell proteins were extracted on ice in lysis buffer (PBS containing 0.5% sodium deoxycholate, 1% NP40, 0.1% SDS, 1 mM sodium orthovanadate, 1 mM PMSF, 1 μg/mL of pepstatin A, and Aprotinin). Briefly, the cells were washed in PBS at 4 °C and scraped on ice into 2–3 ml of ice-cold cell lysis buffer. The cell lysates were then centrifuged at 15,000× *g* at 4 °C to remove the insoluble material and the protein concentrations were determined via Bradford protein assay, as directed by the manufacturer (Thermo-Fisher Scientific, Waltham, MA, USA). The protein extracts of known concentrations were reduced in 1 × reducing SDS-PAGE sample buffer (62.5 mM Tris HCl pH 6.8, 2.5% SDS, 0.002% bromophenol blue, 5% β-mercaptoethanol, and 10% glycerol) and subjected to reducing SDS-PAGE–Western blotting, as previously described [26,42,51]. Briefly, the reduced and denatured proteins separated by SDS-PAGE were transferred to Hybond C-Extra nitrocellulose membranes (GE Healthcare Life Science, Amersham, UK) and the membranes were air-dried. The non-specific protein binding sites on dried membranes were blocked with blocking solution (5% non-fat milk in 15 mM NaCl, 10 mM Tris-HCl, 10% Tween 20, pH8.0) for 2 h at room temperature and the membranes were then incubated overnight with specific primary antibodies diluted in blocking solution at 4 °C with oscillation. The primary antibody dilutions were 1:500 for the anti-TrkA antibody (B3), 1:1000 for anti-Y490 phosphorylated TrkA (9141), 1:1000 for anti-phosphorylated SER 473 Akt (4060) and anti-calmodulin (sc-137079) antibodies, and 1:3000 for the anti-Akt antibody (9272). Following the incubation, the membranes were washed extensively in blocking solution and incubated for a further 1 h at room temperature with appropriate horseradish peroxidase (HRP)-conjugated goat anti-rabbit or rabbit anti-mouse antibodies and secondary antibodies and diluted to 1:2000 in blocking solution. The membranes were then washed extensively in PBS at room temperature and their immunoreactivity was revealed using an Amersham ECL Western blotting detection kit, as directed (Cytiva, Amersham, UK), in an ImageQuant™ LAS 4000 (GE Healthcare) image analyzer. The images were saved as Jpeg files and analyzed densitometrically using ImageJ software (http://imagej.nih.gov/ij/). The Western blots were performed in duplicate and the experiments were repeated a minimum of two times.

### 4.6. Calmodulin/TrkAIII Co-Immunoprecipitation

The cell extracts in lysis buffer (PBS containing 0.5% sodium deoxycholate, 1% NP40, 0.1% SDS, 1 mM sodium orthovanadate, 1 mM PMSF, 1 μg/mL of pepstatin A, and Aprotinin) (1 mg) were pre-cleared via 2 h of incubation with 1 µg/mL of pre-immune IgG and 20 μL/mL of pre-washed Fast Flow Protein A Sepharose at 4 °C with rotation, then the Protein A-Sepharose and IgG were subsequently removed by centrifugation at 15,000 rpm for 5 min at 4 °C. The pre-cleared extracts were incubated overnight with anti-CaM antibody diluted to 1:200 in lysis buffer at 4 °C with rotation. Following incubation, 20 μL of Protein A Sepharose in cell lysis buffer was added and the reactions were incubated for 60 min, with rotation at 4 °C. The Protein A-Sepharose/IgG conjugates were then collected by centrifugation (15,000 rpm for 5 min), washed 3 times in 1ml of lysis buffer, re-centrifuged, reduced in SDS-PAGE reducing sample buffer (62.5 mM Tris HCl pH 6.8, 2.5% SDS, 0.002% bromophenol blue, 5% β-mercaptoethanol, and 10% glycerol), and subjected to reducing SDS-PAGE–Western blotting. CaM and TrkA’s immunoreactivity rates were detected using anti-CaM (1:1000 dilution) and anti-TrkA (1:500 dilution) antibodies and the resulting immunoreactivity was compared to appropriate pre-immune IgG immunoprecipitation controls. The co-immunoprecipitation experiments were performed in duplicate and repeated.

### 4.7. Indirect Immunofluorescence (IF)

For the indirect IF testing, the cells grown to sub-confluence on Nunc glass chamber slides (Sigma-Aldrich, St Louis, MI, USA) were washed in PBS, fixed in formalin 10% *v*/*v,* and permeabilized in 100% ice-cold methanol (−20 °C). The fixed, permeabilized cells were incubated for 1 h in blocking solution (1% bovine serum albumin in PBS-0.03% TX100), then incubated for 2 h with primary anti-TrkA and antiY490 phosphorylated antibodies at 1:100 dilution in blocking solution and at room temperature. Following incubation, the chamber slides were washed extensively in PBS and incubated for 1 h with relevant Alexa Fluor 488-labeled donkey anti-rabbit and Alexa Fluor donkey anti-mouse secondary fluorochrome-conjugated antibodies diluted to 1:500 in blocking solution at room temperature. Following incubation, the slides were washed extensively in PBS, mounted with ProLong^TM^ Gold anti-fade reagent containing nuclear DAPI stain, observed under a Zeiss Axioplan 2 fluorescence microscope with a digital camera and Leica M500 Image Manager software, and digitally photographed. The IF experiments were repeated a minimum of two times.

### 4.8. Statistical Analysis

The data were analyzed using Student’s *t*-test (https://www.graphpad.com/quickcalcs/ttest1.cfm), and statistical significance was associated with probabilities of ≤0.05.

## Data Availability

The data presented in this study are available from the corresponding author upon reasonable request.
